# The SARS-CoV-2 S2′-helix compared to stem helix confers higher genetic barrier to antibody resistance

**DOI:** 10.1371/journal.ppat.1014497

**Published:** 2026-08-04

**Authors:** Xuanjia Wang, Shitong Qiao, Zhiheng Bao, Jiaxiu Bai, Chunyan Yi, Qiang Ding, Xiaoyu Sun

**Affiliations:** 1 Shanghai Institute of Infectious Disease and Biosecurity, Fudan University, Shanghai, China; 2 Center for Infection Biology, School of Basic Medical Sciences, Tsinghua University, Beijing, China; 3 Key Laboratory of Multi-Cell Systems, Center for Excellence in Molecular Cell Science, Shanghai Institute of Biochemistry and Cell Biology, Chinese Academy of Sciences, Shanghai, China; UBA: Universidad de Buenos Aires, ARGENTINA

## Abstract

Understanding the potential for resistance to antibodies targeting conserved epitopes on the coronavirus spike protein is essential for developing broad-spectrum antivirals. The S2′-helix and stem helix represent two key conserved epitopes across multiple coronaviruses, with the S2′-helix being broadly conserved throughout the coronavirus subfamily and the stem helix primarily conserved among betacoronaviruses. Here, we demonstrate that the S2′-helix in SARS-CoV-2 possesses a higher genetic barrier to antibody resistance than the stem helix. Potent escape mutations in the stem helix, including D1153G, Y1155S, F1156L and F1156V, were selected under antibody pressure, and variants such as S1147L, E1151D, D1153G, D1153H, D1153Y and Y1155H were frequently identified in naturally circulating strains. These mutations completely abolished neutralization by some stem helix-targeting antibodies. In contrast, under pressure from S2′-helix-targeting antibodies, we did not detect clear viral escape. Only one naturally occurring mutation in the S2′-helix, L822F, was observed at considerable frequency; it weakly or mildly reduced but did not abolish neutralizing activity. Furthermore, combination therapy with S2′-helix-targeting antibodies synergistically suppressed the emergence of escape mutants selected by stem helix-directed antibodies. Our findings underscore the S2′-helix as a promising target for the design of broadly protective coronavirus therapeutics with a reduced risk of viral escape.

## Author summary

The development of broadly protective therapeutics against coronaviruses is hampered by the potential for viral escape. It is critical to evaluate the potential impact of resistance mutations in the conserved S2 domain of spike, as numerous vaccine and therapeutic efforts are currently focused on S2. This study demonstrates the S2′-helix, a highly conserved region of the spike protein, is an epitope with a high genetic barrier to antibody resistance, in contrast to the stem helix epitope. However, combination with S2′-helix-targeting antibodies synergistically suppressed the emergence of escape mutants selected by stem helix-directed antibodies. These findings provide a mechanistic basis for designing broadly protective coronavirus therapeutics capable of overcoming viral resistance.

## Introduction

The spike (S) glycoprotein serves as the principal antigen of severe acute respiratory syndrome coronavirus 2 (SARS-CoV-2) and has undergone extensive antigenic drift since the onset of the COVID-19 pandemic [1–6]. S protein is composed of an N-terminal domain (NTD), a receptor-binding domain (RBD), and an S2 domain [7,8]. The RBD domain is immunodominant but highly susceptible to mutations [2–6]. In contrast, the S2 domain presents an attractive target for antibody development due to its higher conservation across multiple coronaviruses compared with RBD and NTD regions, suggesting a greater genetic barrier to resistance under selective pressure. Antibodies targeting the S2 domain inhibit viral-host membrane fusion, thereby blocking viral entry into host cells [9–16]. The identification and characterization of broadly neutralizing antibodies (bnAbs) against the S2 domain have provided critical insights into universal coronavirus vaccine and therapeutic design [17–19].

**Fig 1:**
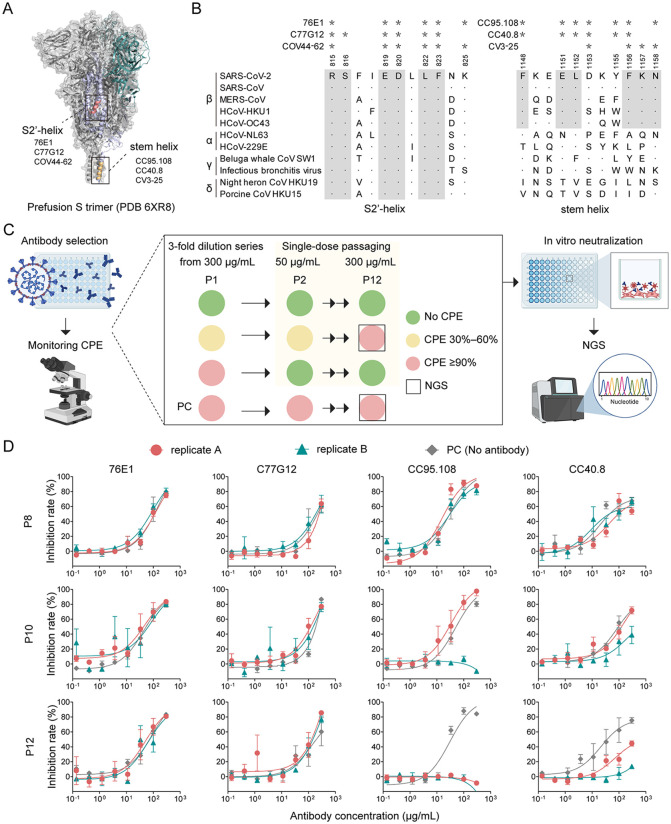
The S2′-helix exhibits a higher barrier to antibody resistance than the stem helix under antibody pressure in vitro. **(A)** Structural localization of S2-targeting bnAbs on the SARS-CoV-2 spike trimer (PDB: 6XR8). Two protomers are shown in gray, and the third protomer is colored with S1 in light teal and S2 in blue white. Conserved epitopes are highlighted: S2′-helix (salmon) targeted by 76E1, C77G12, and COV44-62; stem helix (light orange) targeted by CC95.108, CC40.8, and CV3-25. **(B)** Sequence alignment of the S2′-helix and stem helix regions across alpha-, beta-, gamma- and deltacoronaviruses. Critical antibody-contact residues are marked with an asterisk (*), dots indicate residues identical to those in SARS-CoV-2. **(C)** Schematic of the escape mutant screening workflow. At passage 1, an initial antibody dose-dependent neutralization was performed using 3-fold serial dilutions starting at 300 μg/mL. Viruses were then serially passaged 11 times, with one antibody concentration used at each passage and increased stepwise across passages. Viral populations in black boxes were selected for next-generation sequencing (NGS) to identify mutations. A no-antibody control was sequenced from each passage to monitor for tissue culture adaptations. CPE was scored as follows: green (none), yellow (30–60%), pink (≥90%). Created in BioRender. Xie, **W.** (2026) https://BioRender.com/n9fjkyf **(D)** Neutralization resistance of viruses from passages 8, 10 and 12 across two independent replicates **(A and B)**. Data are shown as mean ± SEM and are representative of two independent experiments.

To date, two neutralizing antibody epitopes within S2, the stem helix and S2′-helix, have been reported [9–11,13–16]. The stem helix (residues 1147–1158, SARS-CoV-2 Wuhan-Hu-1 numbering) is highly conserved among betacoronaviruses. Multiple stem helix-targeting antibodies, including CC95.108 [9], CC40.8 [10] and CV3-25 [11], have been reported (Fig 1A and 1B). A Cryo-electron tomography (cryo-ET) study revealed that these antibodies inhibit refolding of the S2 prehairpin intermediate [12]. The S2′-helix (residues 815–825), which contains the functionally critical S2′ cleavage site, demonstrates even greater conservation than the stem helix, conferring remarkable neutralizing breadth against alpha-, beta-, gamma-, and deltacoronaviruses [19]. We and other groups have previously identified the S2′-helix-targeting antibodies, including 76E1, COV44-62 and C77G12 [13–15], which exhibited pan-coronavirus reactivity against seven human coronaviruses: SARS-CoV-2, SARS-CoV, MERS-CoV, HCoV-OC43, HCoV-HKU1, HCoV-229E, and HCoV-NL63 (Fig 1A and 1B). This cryptic epitope is partially occluded in the prefusion S conformation but becomes accessible following angiotensin-converting enzyme 2 (ACE2) receptor engagement [14,20].

Resistance mutations pose significant challenges for antiviral and vaccine development [21–24]. Despite both epitopes being conserved among SARS-CoV-2 variants under monitoring (VUMs), variants of interest (VOIs), and variants of concern (VOCs) (S1 Fig), several studies have selected resistance mutations in the stem helix through virus passaging [16,25]. Others have identified resistance mutations in the S2′-helix by deep mutational scanning [26]. However, the extent of resistance to stem helix- and S2′-helix-targeting bnAbs has not been comparatively analyzed. Here, we systematically compare the genetic barriers to resistance for bnAbs targeting these two S2 epitopes. Our findings demonstrate that the S2′-helix confers a substantially higher genetic barrier to antibody resistance than the stem helix in SARS-CoV-2, establishing it as a promising target for pan-coronavirus countermeasure design with a reduced risk of viral escape.

## Results

### Antibody-driven selection of SARS-CoV-2 escape mutants

To comparatively evaluate escape barriers between the S2′-helix and stem helix, we selected stem helix (CC95.108 and CC40.8)- and S2′-helix (76E1 and C77G12)-targeting antibodies for subsequent viral escape mutant screening (Fig 1A). Key epitope residues, previously identified by cryo-electron microscopy (cryo-EM), are largely overlap among antibodies targeting the same epitope (Fig 1B). Replication-competent VSV-Spike pseudovirus (rVSV-S), in which the native VSV glycoprotein was replaced with the SARS-CoV-2 spike (S) protein, was used for viral passage under antibody selective pressure. An initial antibody dose-dependent neutralization assay was performed in the first passage (P1). Viral supernatants were then passaged eleven times in the presence of progressively increasing concentrations of each individual antibody (Fig 1C). Neutralization-resistant mutants readily emerged under specific antibody concentrations. During selection, viral populations were passaged in duplicate as independent replicates (A and B). An untreated group served as a positive control.

To confirm the emergence of escape mutants, we selected viral populations from passages 8, 10, and 12 for antibody neutralization assays based on initial cytopathic effects (CPE) monitoring for emerging resistance. P8, which showed no evidence of escape, was included as a pre-escape control. In contrast, P10 and P12 were selected because resistance emerged in these passages. Antibodies 76E1 and C77G12 maintained similar neutralizing efficacy against P8, P10, and P12 viruses from both replicates compared to the original virus. In contrast, viruses from replicate A at P12 and replicate B at both P10 and P12 exhibited high-level resistance to CC95.108, with complete loss of neutralizing activity. Viruses from both replicates at P12 escaped neutralization by CC40.8 (Fig 1D).

These results indicate that the S2′-helix exhibits a substantially higher escape barrier to antibody resistance compared to the stem helix under in vitro antibody pressure.

### Validation of antibody-selected escape mutations

**Fig 2:**
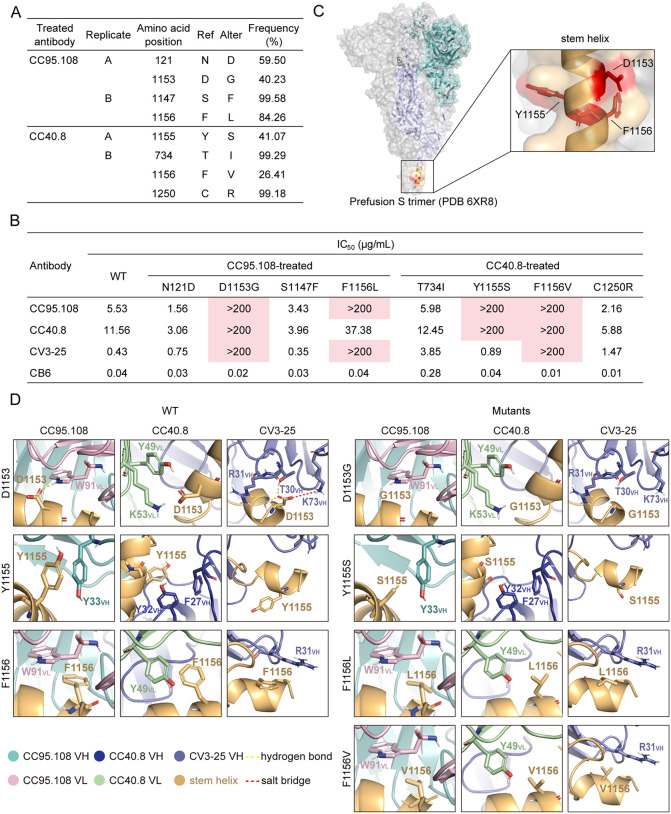
Validation of antibody-selected escape mutations in stem helix. **(A)** NGS results of CC95.108- and CC40.8-selected escape mutations within stem helix across two independent replicates. Mutations enriched under antibody pressure and absent in passage-matched controls are shown. **(B)** Neutralization IC_50_ values of stem helix–targeting antibodies against SARS-CoV-2 pseudoviruses carrying individual escape mutations, measured in 293T-ACE2 cells. The RBD-targeting antibody CB6 serves as a control. Data shown are representative of two independent experiments. **(C)** Structural localization of key escape mutation positions within the prefusion spike (PDB: 6XR8). **(D)** Structural models of SARS-CoV-2 stem helix (light orange) harboring D1153G, Y1155S, F1156L or F1156V mutations in complex with CC95.108 (heavy chain: light teal, light chain: light pink), CC40.8 (heavy chain: deep blue, light chain: pale green) or CV3-25 heavy chain (slate). Left panels: wild-type residues; right panels: mutant residues.

To further validate antibody-selected escape mutations, we performed next-generation sequencing on passage 12 rVSV-S populations (S1 Table). Mutations were selected for subsequent characterization using three criteria: (1) ≥ 20% frequency in antibody-treated samples, (2) absence in passage-matched negative controls, and (3) exclusion of synonymous changes. Under CC95.108 pressure, we identified mutations N121D, D1153G, S1147F, and F1156L. Conversely, CC40.8 selection yielded distinct mutations Y1155S, T734I, F1156V and C1250R (Fig 2A). Notably, positions 1147, 1153, 1155 and 1156 localize to the stem helix region.

The neutralizing activity of three stem helix-targeting antibodies (CC95.108, CC40.8, and CV3-25) was further evaluated against pseudoviruses carrying single-point mutations. An RBD-targeting antibody, CB6, was included as a control [27]. The D1153G mutation conferred broad resistance to all three stem helix-targeting antibodies, while remaining susceptible to CB6. The F1156L mutation completely abolished neutralization by CC95.108 and CV3‑25 but only slightly reduced that of CC40.8. The Y1155S mutation led to a complete loss of neutralization by CC95.108 and CC40.8 but partially reduced neutralization by CV3-25. The F1156V mutation abolished neutralization by all three stem helix-targeting antibodies. CC40.8 retained partial neutralizing activity against F1156L but was significantly impaired against F1156V, highlighting antibody-specific escape profiles. Although S1147 lies within the epitope, mutation to phenylalanine (F) did not affect neutralization, confirming its non-critical role in antibody binding (Fig 2B). All identified escape residues are located within the stem helix epitope (Fig 2C). Since stem helix antibodies neutralize viruses primarily through inhibition of membrane fusion, we accordingly assessed their capacity to inhibit fusion mediated by mutant spike proteins. Consistent escape patterns were observed in the membrane fusion inhibition assay: for the D1153G, F1156V, and F1156L mutants, all three stem helix-targeting antibodies showed reduced or abolished membrane fusion inhibitory activity. For Y1155S, both CC95.108 and CC40.8 exhibited reduced fusion inhibition. One exception was CV3-25, which lost fusion inhibitory activity against Y1155S, a finding that diverged from its neutralization profile; this discrepancy may reflect differences between cell–cell fusion and virus–cell fusion assays (S2 Fig). Overall, these data demonstrate that, despite targeting the same epitope (stem helix), different antibodies select distinct resistance mutations, with D1153, Y1155, and F1156 acting as hotspots that drive escape.

We further investigated the structural basis of antibody-selected escape mutations through structural alignment and in silico mutagenesis. Given the high conservation of coronavirus stem-helix epitopes, we modeled the interaction between CC95.108 and the SARS-CoV-2 spike through structural alignment. The CC95.108-bound HCoV-HKU1 stem helix complex (PDB: 8DGW) was structurally aligned to the SARS-CoV-2 stem helix from the CC25.106-bound structure (PDB: 8DGU), enabling analysis of binding determinants. Other published structures of antibody-bound stem helix complexes, including those of CC40.8 (PDB: 7SJS) and CV3-25 (PDB: 7RAQ), were also used for further in silico mutagenesis followed by structural analysis using the Schrödinger Suite. The D1153G substitution completely abolishes side chain-mediated interactions with all three antibodies (Fig 2D). Specifically, it eliminates a hydrogen bond with W91 in the light chain of CC95.108; a hydrogen bond with Y49 and a salt bridge with K53 in the light chain of CC40.8; and hydrogen bonds with T30/R31 and a salt bridge with K73 in the heavy chain of CV3-25. These structural observations explain the loss of neutralization by all three antibodies caused by the D1153G mutation. Furthermore, the Y1155S mutation abolishes π-stacking interactions with Y33 in the heavy chain of CC95.108 and also with F27 and Y32 in the heavy chain of CC40.8. In contrast, this mutation does not affect binding to CV3-25, as Y1155 does not directly interact with this antibody, clarifying why neutralization by CV3-25 remains largely unaffected. The residue F1156 forms π-stacking interactions with W91 in the light chain of CC95.108 and Y49 in the light chain of CC40.8, as well as a cation-π interaction with R31 in the heavy chain of CV3-25. Mutation from F to V disrupts all these interactions, thereby conferring full resistance to all three antibodies. However, mutation from F to L at position 1156 only disrupts neutralization by CC95.108 and CV3-25 via the loss of π-stacking and cation-π interactions, respectively, but marginally reduces the neutralizing potency of CC40.8. This differential effect may be explained by the bulkier side chain of L compared with that of V, which could help maintain certain hydrophobic interactions with CC40.8 despite the absence of π-stacking.

### Natural occurrence of mutations in the stem helix epitope

**Fig 3:**
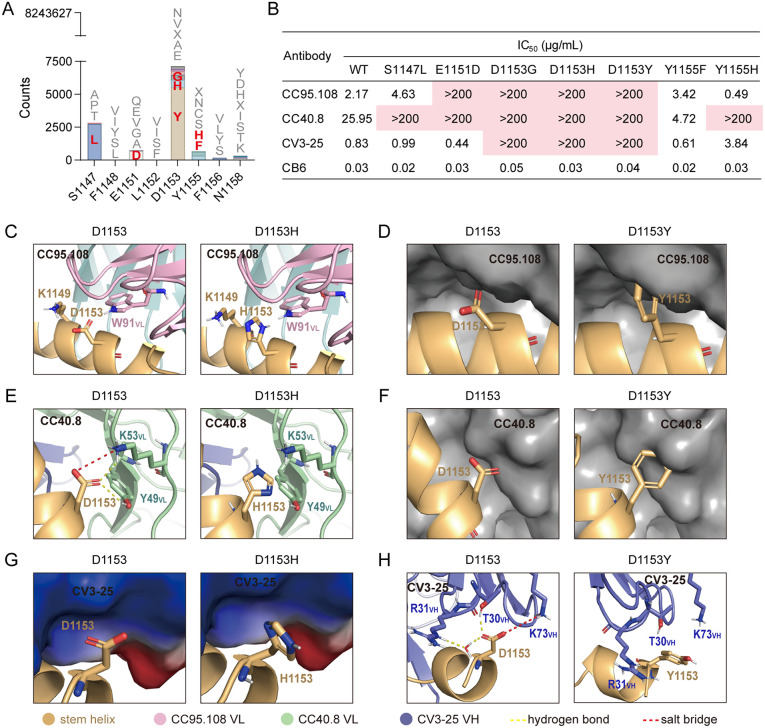
Natural occurrence of mutations in the stem helix epitope. **(A)** Mutation frequency within stem helix residues based on 8,243,627 SARS-CoV-2 sequences from China National Center for Bioinformation (CNCB) as of October 22, 2024. Substitutions with > 300 occurrences are highlighted in bold red, and others in gray. Seven mutations met this threshold within the stem helix epitope. **(B)** Neutralization IC_50_ values of stem helix-targeting antibodies against SARS-CoV-2 pseudoviruses carrying individual escape mutations, measured in 293T-ACE2 cells. The RBD-targeting antibody CB6 serves as a control. Mutations D1153G, D1153H, and D1153Y abolish neutralization by all three stem helix antibodies. Data shown are representative of two independent experiments. **(C–H)** Structural models of the SARS-CoV-2 stem helix (light orange) carrying D1153H **(C, E, G)**, or D1153Y **(D, F, H)** mutations in complex with CC95.108 light chain (light pink), CC40.8 light chain (pale green), or CV3-25 heavy chain (slate). Antibodies are shown in cartoon representation in **(C)**, **(E)**, and **(H)**, and as molecular surfaces in **(D)**, **(F)**, and **(G)**. In **(G)**, the electrostatic surface potential is shown.

To comparatively assess natural genetic barriers between S2′-helix and stem helix epitopes, we systematically analyzed mutation frequencies at key epitope residues. Using 8,243,627 SARS-CoV-2 sequences from China National Center for Bioinformation (as of 22 October 2024), we identified mutations with > 300 occurrences [28–31]. These sequences were collected from individuals worldwide, and clinical metadata regarding vaccination status prior to infection were not available for this dataset. Multiple mutations within the stem helix met this threshold. The most frequently mutated residue is D1153, with the D1153Y mutation detected in 5,524 SARS-CoV-2 sequences, followed by D1153H (*n* = 592) and D1153G (*n* = 362). Secondary hotspots include Y1155 (Y1155H, *n* = 349; Y1155F, *n* = 304) and E1151 (E1151D, *n* = 702). Given the observed S1147F mutation in preliminary experiments, we specifically included the S1147L variant (*n* = 2,763) in our subsequent analysis (Fig 3A). Pseudovirus neutralization assays revealed distinct escape patterns: (1) D1153G/H/Y conferred pan-resistance to all three tested anti-stem helix antibodies; (2) S1147L showed complete escape from CC40.8; (3) E1151D was resistant to both CC95.108 and CC40.8; and (4) Y1155H escaped neutralization by CC40.8 and CV3-25. CB6 maintained full neutralization potency against all tested variants and was included as the control (Fig 3B). Notably, D1153G/H/Y variants showed a broad resistance profile, which was also confirmed in the membrane fusion inhibition assay (S3 Fig). These data highlight a higher natural mutation frequency within the stem helix region than within the S2′-helix, and residue D1153 is critical for mediating escape from stem helix-targeting antibodies. To investigate the structural mechanisms of antibody escape by D1153 variants (G/H/Y), we conducted structural alignment and in silico mutagenesis. As described above, the D1153G mutation abolishes all side chain-mediated interactions with the three antibodies (Fig 2D). Substitution histidine (D1153H) introduces a positive charge at this position, resulting in unfavorable interactions with surrounding residues: it repels K1149 in the stem helix and light chain W91 in CC95.108 (Fig 3C), disrupts a hydrogen bond with Y49 and a salt bridge with K53 in CC40.8 (Fig 3E), and causes charge incompatibility-driven repulsion with CV3-25 (Fig 3G). In contrast, the tyrosine substitution (D1153Y) leads to steric hindrance within the binding interfaces due to its bulky side chain: it clashes with CC95.108 and CC40.8 (Fig 3D and 3F), and compromises hydrogen bonds with T30/R31 and a salt bridge with K73 in CV3-25 (Fig 3H). Collectively, these structural insights establish D1153 as a critical escape position for anti-stem helix antibodies, mechanistically explaining the loss of neutralization by all three anti-stem helix antibodies.

### Natural occurrence of mutations in the S2′-helix epitope

**Fig 4:**
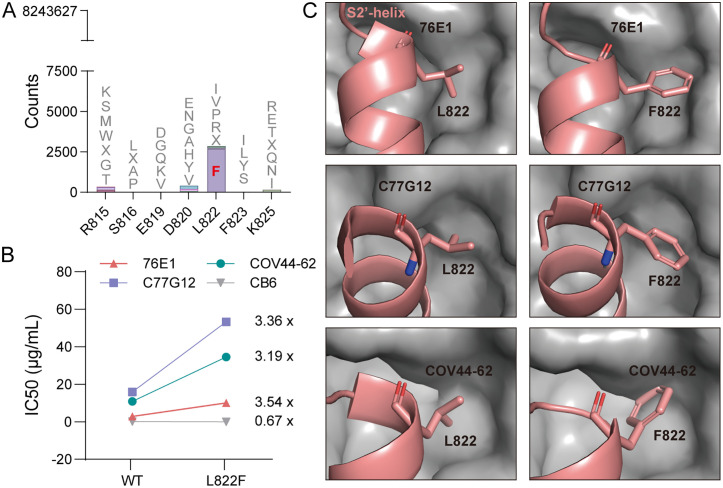
Natural occurrence of mutations in the S2′-helix epitope. **(A)** Mutation frequency of the key epitope residues within the S2′-helix based on 8,243,627 SARS-CoV-2 sequences from the China National Center for Bioinformation (CNCB) as of October 22, 2024. Substitutions with > 300 occurrences are shown in bold red, and others in gray. One mutation met this threshold within the S2′-helix. **(B)** Neutralization IC_50_ values of S2′-helix–targeting antibodies against SARS-CoV-2 pseudoviruses carrying the L822F mutation, measured in 293T-ACE2 cells. The RBD-targeting antibody CB6 serves as a control. The L822F mutation weakly or mildly reduces but does not abolish neutralizing activity. Data shown are representative of two independent experiments. **(C)** Structural models of the SARS-CoV-2 S2′-helix (salmon, cartoon) bearing the L822F mutation in complex with 76E1, C77G12, or COV44-62 (gray, surface). Left: wild-type L822; right: mutant F822.

In contrast, only one S2′-helix mutation met the threshold as described above: L822F (*n* = 2,698) (Fig 4A). Pseudovirus neutralization assays revealed that L822F conferred weak or mild resistance to three anti-S2′-helix antibodies (76E1, C77G12 and COV44-62), increasing half-maximal inhibitory concentration (IC_50_) values by ~3-fold relative to the wild-type (Fig 4B). Consistently, L822F had little effect on the membrane fusion inhibition by these antibodies (S4 Fig). Structural analysis of published complexes (76E1: 7X9E; C77G12: 7U0A; COV44-62: 8D36) demonstrated that the bulkier side chain of F822 causes minor steric clashes with antibody paratope residues, which may subtly impair neutralization (Fig 4C).

Additionally, we found that L822F exhibited reduced spike expression and impaired spike-mediated membrane fusion activity compared with WT (S5 Fig). These findings suggest that L822F may compromise viral fitness and thereby limit its prevalence.

### Natural mutation frequencies in the S2′-helix and stem helix under immune pressure

To further assess the frequency of mutations under different population-level immune pressures, an additional set of SARS-CoV-2 sequences was downloaded from the GISAID database. Sequences collected on or before 31 December 2020 (n = 525,958) were assigned to the low population immune pressure group, representing the pre-vaccine or minimally vaccine-exposed period, as the first WHO Emergency Use Listing for a COVID-19 vaccine was issued on 31 December 2020 [32]. Sequences collected between 1 July 2022 and 31 December 2024 (n = 148,957) were assigned to the high population immune pressure group, representing a period characterized by widespread global vaccination and repeated SARS-CoV-2 circulation. By late June 2022, more than 60% of the global population had completed primary COVID-19 vaccination [33]. Sequences collected between 1 January 2021 and 30 June 2022 were considered to represent a transition period and were excluded from the comparison.

**Fig 5:**
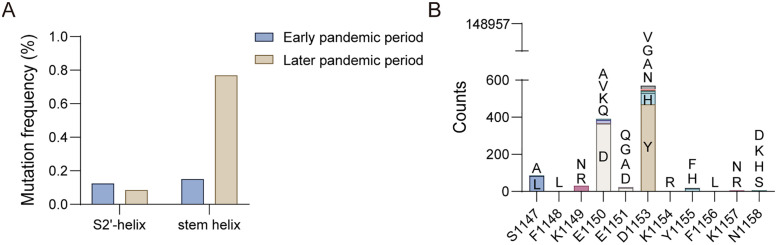
Natural mutation frequencies in the S2′-helix and stem helix epitopes under immune pressure. **(A)** Comparison of mutation frequencies in the S2′-helix and stem helix epitopes between the early pandemic (low immune pressure; on or before December 31, 2020; n = 525,958) and later pandemic (high immune pressure; 1 July 2022 to 31 December 2024; n = 148,957) periods. Data were retrieved from the GISAID database. **(B)** Mutational profile within the stem helix epitope during the later pandemic period. Amino acid substitutions observed at the indicated positions are shown.

Using this dataset, we compared the mutation frequencies within the S2′-helix and stem helix epitopes between the low and high population immune pressure groups. Our analysis showed that during the early pandemic period (low immune pressure), mutation frequencies in both the S2′-helix and stem helix epitopes remained low. In contrast, during the later pandemic period (high immune pressure), the stem helix epitope exhibited a significantly elevated mutation frequency compared with that of the S2′-helix epitope (Fig 5A). Furthermore, we summarized mutations within the stem helix epitope identified during the later pandemic period (Fig 5B). Among these, residue 1153 emerged as a mutational hotspot (Fig 5B), with substitutions such as D1153Y, D1153H and D1153G also identified in the 8,243,627 naturally occurring SARS-CoV-2 sequences analyzed earlier in our study. Although the E1150D mutation is also prevalent, this residue is not a critical binding site for stem helix antibodies.

Collectively, these additional data further support the conclusion that the S2′-helix exhibits a higher genetic barrier to variation than the stem helix under immune pressure.

### Synergistic suppression of stem helix escape mutants by S2′-helix-targeting antibody cocktails

**Fig 6:**
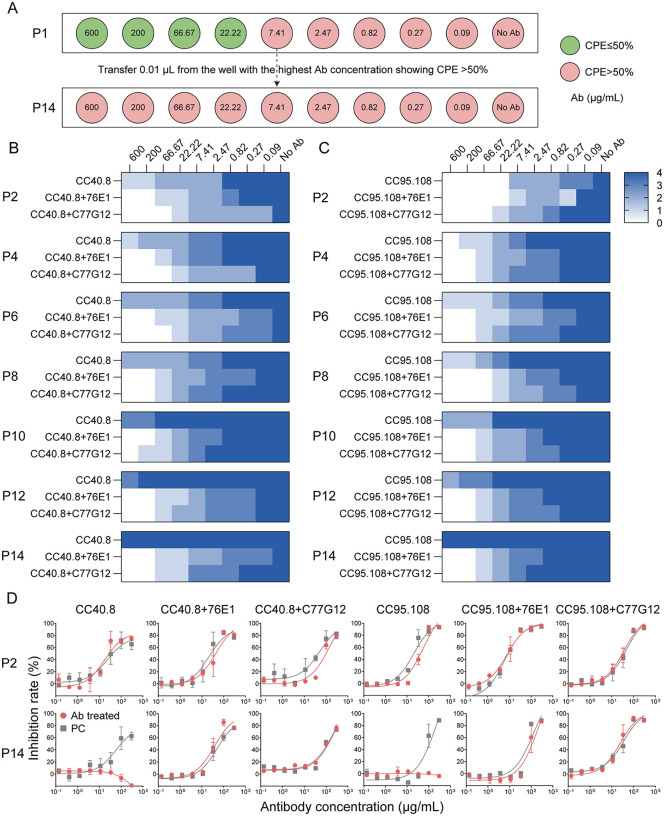
Synergistic suppression of stem helix escape mutants by S2′-helix antibody cocktails. **(A)** Schematic of the escape mutant screening protocol using the rVSV-S system. An initial antibody dose-response neutralization was conducted at passage 1 (P1) using serially diluted antibodies, as indicated in the schematic. Viral replication was assessed by manually scoring CPE. Supernatants from wells showing >50% CPE under the highest antibody concentration were collected for subsequent passaging. P1 supernatants were serially passaged 13 times under continued antibody selection in fresh Vero E6-ACE2 cells. A no-antibody control was included at each passage to monitor tissue culture adaptations. CPE scoring: green (≤50%), pink (>50%). **(B-C)** Assessment of CPE during serial passaging under antibody selection. CPE was graded on a four-point scale (0–4) relative to control wells: 0, no CPE; 1, 0–25%; 2, 25–50%; 3, 50–75%; and 4, 75–100%. **(D)** Neutralization curves of CC40.8 or CC95.108, alone or in combination with C77G12 or 76E1, against viruses from passages 2 and 14 in Vero E6-ACE2 cells. No-antibody control viruses from corresponding passages served as positive controls. Data represent mean ± SEM; results are representative of two independent experiments.

Antibody cocktails represent a key therapeutic strategy to mitigate viral escape [34]. To evaluate whether combination with S2′-helix-targeting antibodies prevents viral escape from stem helix-targeting antibodies, we performed antibody-driven selection of SARS-CoV-2 escape mutants under three conditions using the rVSV-S system: a single stem helix-targeting antibody (CC40.8 or CC95.108); a cocktail of a stem helix-targeting antibody and the S2′-helix-targeting antibody (76E1); and a cocktail of a stem helix-targeting antibody and another S2′-helix-targeting antibody (C77G12). Viral replication was assessed by monitoring virus-induced cytopathic effect (CPE). Supernatants from wells with >50% CPE at maximal antibody concentration were passaged (Fig 6A). In the single-antibody groups, CPE intensity progressively increased through serial passages. In contrast, antibody cocktails maintained stable CPE levels (Fig 6B and 6C). After 14 selection rounds, resistant viruses emerged in single stem helix antibody groups, while viral escape was suppressed in cocktail groups. Further neutralization assays against passage 14 viruses confirmed complete resistance to stem helix antibodies in single-selection groups, while cocktail-selected viruses retained full sensitivity to both antibody classes (Fig 6D). These results demonstrate that combining stem helix-targeting antibodies with S2′-helix antibodies synergistically suppresses the emergence of escape mutants selected by stem helix-targeting antibodies, highlighting the important potential of S2′-helix-targeting antibodies in countering such escape mutations.

## Discussion

The stem helix and S2′-helix represent two key epitopes in S2 targeted by bnAbs capable of neutralizing multiple coronaviruses. Previous studies on coronavirus bnAbs have not systematically addressed whether bnAbs targeting the two epitopes exhibit varying propensities to selected for resistance mutations.

Here, we demonstrate that the S2′-helix possesses a significantly higher genetic barrier to resistance, both under in vitro antibody pressure and in naturally circulating strains, compared to the stem helix. Consequently, S2′-helix-targeting bnAbs may more effectively suppress viral escape than those directed against the stem helix.

Furthermore, the S2′-helix exhibits high conservation across all four major coronavirus genera (alpha-, beta-, gamma-, and deltacoronaviruses), whereas the stem helix is primarily conserved among betacoronaviruses. This broader phylogenetic conservation makes the S2′-helix a more attractive target for the design of broadly protective vaccines and antivirals [19].

The S2 domain is immunologically subdominant. Neutralizing antibodies targeting this region are relatively uncommon and therefore exert limited selective pressure on currently circulating viral strains. However, if S2-directed bnAbs are deployed globally, immune pressure on this region will increase. Previous studies have reported that mutations D1153G and F1156L confer resistance to the stem helix-targeting antibody S2P6 [16]. In our study, we identified a series of resistance mutations within the stem helix under in vitro antibody pressure, including D1153G, Y1155S, F1156L, and F1156V, as well as naturally occurring variants such as S1147L, E1151D, D1153G, D1153H, D1153Y, and Y1155H. Notably, these mutations led to a complete loss of neutralizing activity against some antibodies targeting the stem helix. In contrast, escape mutations within the S2′-helix appear to be less frequent. Previous work using deep mutational scanning identified that the F823Y mutation confers resistance to both COV44-62 and COV44-79 antibodies [26]. However, this variant is rarely observed among naturally circulating strains (Fig 4A). In our study, only one mutation in the S2′-helix, L822F, was found at a notable frequency (*n* = 2698). This mutation weakly reduced but did not abolish neutralization by three tested antibodies. Additionally, L822F exhibited reduced spike expression and decreased spike-mediated membrane fusion activity relative to WT. Notably, under pressure from S2′-helix-targeting antibodies, we did not detect clear viral escape, further suggesting that mutations within the S2′-helix may confer a fitness disadvantage. In contrast, the emergence of D1153G, Y1155S, F1156L and F1156V within the stem helix region as escape mutations under in vitro antibody selection pressure suggests that these substitutions are at least tolerated and are unlikely to impose a major fitness cost. It is critical to evaluate the potential impact of such mutations, as numerous vaccine and therapeutic efforts are currently focused on the S2 region [17,18,35].

Although the stem helix region exhibits a higher propensity for resistance mutations, we show that antibody cocktails incorporating S2′-helix-targeting bnAbs can prevent or overcome such escape.

We acknowledge several limitations in this study. First, while pseudovirus platforms are widely used to evaluate S2-targeting antibodies and peptides, with reported potencies similar to or not significantly different from those obtained with authentic SARS-CoV-2 [9,13–16,36], we recognize that authentic virus-based assays remain the gold standard. Moreover, our in vitro rVSV-S -based selection experiments may not fully capture the complexity of viral evolution in vivo, where factors such as heterogeneous receptor expression, cell-type-specific entry pathways, and host immune pressures beyond neutralizing antibodies could influence escape dynamics. Future studies using animal models or clinical samples from antibody-treated individuals will be essential to definitively evaluate the in vivo escape potential of these epitopes. Second, although this study is focused on SARS-CoV-2, our findings may theoretically extend to other coronaviruses, as several stem helix- and S2′-helix-targeting bnAbs have been reported to exhibit cross-neutralizing activity against diverse coronaviruses [9,11,13–16]. Rigorous experimental validation using other coronaviruses will be required to confirm this possibility, which represents an important direction for our future work. Additionally, mutations outside the epitope can also affect neutralization (e.g., H655Y partially impairs neutralization by 76E1 [37]). However, given the unknown extra-epitope influences on stem helix-targeting antibodies and the stronger selective pressure antibodies exert on epitope residues, we restricted the comparative analysis to epitope sites.Despite these limitations, the consistent observation that the S2′-helix exhibits a higher genetic barrier to resistance, demonstrated through both in vitro antibody pressure selection and large-scale sequence analyses of naturally circulating strains, supports the reliability of our main findings. Our conclusions should therefore be interpreted as a comparative assessment of epitope vulnerability under defined experimental conditions, rather than absolute predictions of in vivo therapeutic outcomes.

In summary, the S2′-helix represents an ideal target for the design of broad-spectrum antiviral strategies due to its high conservation across coronaviruses and high barrier to antibody resistance. Targeting this epitope may enable the development of next-generation countermeasures with broad coverage and reduced risk of viral escape.

## Materials and methods

### Cells and viruses

Vero E6 cells expressing human ACE2 (Vero E6-ACE2), HEK293T cells and HEK293T cells expressing human ACE2 (293T-ACE2) were maintained in Dulbecco′s Modified Eagle Medium (DMEM, Gibco) supplemented with 10% fetal bovine serum (FBS) and 1% penicillin/streptomycin at 37°C with 5% CO_2_. ExpiCHO cells (ThermoFisher Scientific) were propagated in respective CD05 expression medium (YOUYI) at 37°C, 8% CO_2_, and shaken at 120 rpm. A replication-competent SARS-CoV-2 S protein-pseudotyped vesicular stomatitis virus (rVSV-S), bearing the spike sequence of the wild-type Wuhan-Hu-1 strain (GenBank accession number MN908947), was propagated in Vero E6-ACE2 cells cultured in DMEM supplemented with 2% FBS. SARS-CoV-2 pseudoviruses were also prepared in-house using HEK 293T cells.

### Preparation of antibodies

To prepare full-length antibodies, the genes encoding the variable heavy (VH) and variable light (VL) domains of the antibodies were codon-optimized for mammalian expression and then cloned into designated vectors: pCAGGS-hIgG1-VH for the heavy chain and either pCAGGS-hIg-Vκ or pCAGGS-hIg-Vλ for the light chain, both incorporating a mouse antibody signal peptide. Gene sequences of antibodies 76E1 (PDB: 7X9E) were prepared in-house, while those of COV44-62 (PDB: 8D36), C77G12 (PDB: 7U0A), CC95.108 (PDB: 8DGW), CC40.8 (PDB: 7SJS) and CV3-25 (PDB: 7RAQ) were obtained from the Protein Data Bank (PDB). Subsequently, the VH and VL plasmids were co-transfected into ExpiCHO cells and cultured for seven days. The antibodies were then purified via Protein A and further refined through size exclusion chromatography.

### Generation of antibody-resistant rVSV-S viruses

Antibody-resistant rVSV-S mutants were selected according to a previously described protocol [38]. Briefly, Vero E6-ACE2 cells were seeded in 96-well plates at 3 × 10⁴ cells/well and cultured overnight at 37°C with 5% CO_2_. A low dose of rVSV-S viruses (30 TCID_50_ per well) was pre-incubated with three-fold serially diluted antibodies with a starting concentration of 300 μg/mL for 1 hour. The virus-antibody mixtures were then transferred to the pre-plated cells and incubated for 48 hours. Two replicate wells were used for each antibody condition, with virus-only controls processed in parallel. Cytopathic effects (CPE) were monitored manually by microscopy [24]. An amount equivalent to 0.01 μL of each sample (passage 1 [P1] virus; 1 μL of a 100-fold dilution) was incubated with 50 μg/mL of antibody and used to infect fresh Vero E6-ACE2 cells. After 2 days of culture, an amount equivalent to 0.01 μL of each sample (1 μL of a 100-fold dilution) of the resulting P2 virus was subjected to another round of selection with an increased concentration of antibody, yielding P3 virus. This selection was performed for several rounds under progressively increasing antibody pressure over passages. The generation of antibody-resistant rVSV-S viruses was determined by antibody-mediated neutralization assays.

### rVSV-S neutralization assays

Each antibody-resistant rVSV-S mutant sample was first titrated to determine viral infectivity using the 50% tissue culture infectious dose (TCID_50_) assay. Neutralization assays were subsequently conducted in 96-well plates using a fixed input of 100 TCID_50_ per well. Vero E6-ACE2 cells were seeded at a density of 3 × 10⁴ cells per well and incubated overnight at 37°C with 5% CO_2_. The passaged rVSV-S was pre-incubated with three-fold serial dilutions of each antibody, starting at 300 μg/mL, for 1 hour at 37°C before being applied to the cell monolayer. After 48 hours of infection, culture supernatants were removed, and cells were fixed with 80% acetone for 30 minutes at room temperature (RT).

The plates were washed three times with PBS containing 0.05% Tween-20 (PBST), then blocked with 1% BSA in PBST for 1 hour at RT. After removing the blocking solution, 50 μL of rabbit anti-SARS-CoV-2 S2 polyclonal antibody (Sino Biological, Cat# 40590-T62) was added to each well and incubated for 1 hour at RT. Following another round of washing, 50 μL of HRP-conjugated Goat Anti-Rabbit IgG(H + L) (Proteintech, Cat# SA00001–2) was added per well and incubated for 40 minutes at RT. After a final wash, 100 μL of TMB substrate solution (Ncmbio, Cat# M30500) was added to each well and incubated for 20 minutes at RT in the dark. The reaction was terminated by adding 50 μL of stop solution per well, and the optical density (OD) at 450 nm was immediately measured using a Spark Multimode Microplate Reader (Tecan). The relative percent inhibition was calculated as follows:


$$\begin{array}{c} \text{Relative inhibition }(\%)\;=\;[({\text{OD}}_{\left(\text{rVSV}-\text{S control}\right)}\;-\;{\text{OD}}_{\left(\text{Test sample}\right)})\;/\;({\text{OD}}_{\left(\text{rVSV}-\text{S control}\right)}\;-\;{\text{OD}}_{\left(\text{mock}-\text{infected control}\right)})]\;\times \text{ 100}\%. \end{array}$$

### Sequencing of antibody-resistant rVSV-S viruses

Viral RNA was extracted using TIANamp Virus RNA Kit (TIANGEN, Cat# DP315-R) and the spike region of rVSV-S was amplified and purified using HiScript lll 1st Strand cDNA Synthesis Kit (Vazyme, Cat# R312-01). Illumina sequencing and library construction were performed at the Shanghai Tanpu Biotechnology Co., Ltd (Shanghai, China). In brief, the NEBNext Ultra II RNA Library Prep Kit (NEB, Ipswich, MA, USA) was used for library construction. After adapter ligation, 10 cycles of PCR amplification were performed to enrich the sequencing target. The libraries were pooled in equimolar amounts, denatured, and diluted to the optimal concentration before sequencing. The Illumina NovaSeq 6000 System (Illumina, San Diego, CA, USA) was used for sequencing to generate 150-bp paired-end reads. As for SNP analysis, high-quality filtered reads were mapped against the reference genome using the Burrows-Wheeler Aligner (v2.04), which also generated a BAM file to calculate the mapping depth and coverage. SNPs were identified using an integrated software Snippy (v4.6.0), which included both single-nucleotide polymorphisms (SNPs) and insertions/deletions (indels). Variants were selected if mapping quality ≥ 40 and depth ≥ 5.

### Pseudovirus production and neutralization

Based on sequencing-derived mutation profiles, pCDNA3.1 plasmids encoding mutant S genes were constructed. These plasmids were then co-transfected with the pNL4–3.Luc.R-E-backbone into HEK293T cells using the HighGene Plus Transfection Reagent (ABclonal, Cat# RM09014P), following the manufacturer′s protocol. 6 hours post-transfection, 50% of the medium was replaced with fresh DMEM. Viral supernatants were harvested at 48 hours, clarified by centrifugation, and aliquoted for subsequent assays. For the neutralization assay, 293T-ACE2 cells were seeded in white 96-well plates at a density of 2.5 × 10⁴ cells per well and cultured at 37°C with 5% CO_2_ overnight. Viruses were diluted in DMEM supplemented with 10% FBS and pre-incubated with three-fold serial dilutions of antibodies at a starting concentration of 200 μg/mL for 1 hour before infecting the pre-plated cells. Following a 48-hour incubation, luciferase activity was quantified using the Bright-Lite Luciferase Assay System (Vazyme, Cat# DD1204-01) according to the manufacturer′s protocol. Luminescence signals were measured using a Spark Multimode Microplate Reader (Tecan) and analyzed using GraphPad Prism 9.5.0 (GraphPad Software, San Diego, CA). Except for the indicated mutations, the amino acid sequences of the spike protein used in the preparation of pseudoviruses were identical to the Wuhan-Hu-1 isolate (NCBI reference sequence: YP_009724390.1). CB6, an anti-SARS-CoV-2 spike RBD antibody, was included as a control. Percent neutralization was derived from relative luminescence reduction compared to untreated virus controls.

### Syncytium inhibition assay

HEK293T cells were seeded in 12-well plates and cultured at 37°C with 5% CO_2_ overnight. The following day, effector and target cells were co-transfected with 1 μg/well plasmids (1:1) to express SARS-CoV-2 Spike and Cre recombinase, or human ACE2 and a loxP-STOP-loxP luciferase reporter, respectively. All transfections were performed using the HighGene Plus Transfection Reagent according to the manufacturer′s protocol. At 24 h post-transfection, effector cells were harvested, incubated with indicated antibodies at 37°C for 1 h and then co-cultured with an equal number of target cells in white 96-well plates for an additional 24h. Following this incubation, luciferase activity was quantified using the Bright-Lite Luciferase Assay System according to the manufacturer′s protocol. Luminescence signals were measured using a Spark Multimode Microplate Reader (Tecan) and analyzed using GraphPad Prism 9.5.0.

### Western blot

HEK293T cells were seeded in 24-well plates and cultured at 37°C with 5% CO_2_ overnight. The following day, the cells in each well were transfected with 1 μg of spike-expression plasmids with HighGene Plus Transfection Reagent according to the manufacturer′s protocol. At 24 h post-transfection, the culture medium was removed, and cells were lysed directly in each well by adding 100 μL of SDS loading buffer containing β-mercaptoethanol. The cells were scraped thoroughly and collected into microcentrifuge tubes, followed by boiling at 95°C for 10 min. Equal volumes of protein samples (20 μL per lane) were loaded onto 7.5% SDS-PAGE gels for electrophoretic separation and then transferred onto PVDF membranes. After blocking with 5% non-fat milk in TBST for 1 h at room temperature (RT), the membranes were incubated with rabbit anti-SARS-CoV-2 S2 polyclonal antibody for 1 h at RT, followed by incubation with HRP-conjugated Goat Anti-Rabbit IgG(H + L) for 1 h at RT. For β-actin detection, membranes were incubated with HRP-conjugated β-actin monoclonal antibody (Proteintech, Cat# HRP-60008) for 1 h at RT. Protein bands were detected using NcmECL Ultra (NCMBiotech, Cat# P10100) and visualized using a chemiluminescence imaging system.

### Sequence alignment

To compare the conservation of S2′-helix and stem helix epitopes, the spike protein sequences of SARS-CoV (GenBank: AAP41037.1), SARS-CoV-2 (GenBank: QHD43416.1), MERS-CoV (GenBank: AFS88936.1), HCoV-NL63 (GenBank: AAS58177.1), HCoV-OC43 (GenBank: AAT84354.1) and HCoV-229E (GenBank: AAG48592.1) were aligned using NCBI protein BLAST suite as shown in Fig 1B.

To identify natural mutations in the epitope residues of S2′-helix and stem helix, variant annotation data from 8,243,627 high-quality SARS-CoV-2 sequences were downloaded from China National Center for Bioinformation (as of 22 October 2024) https://ngdc.cncb.ac.cn/ncov/variation/annotation.

To compare mutation frequencies in the S2′-helix and stem helix epitopes under different population-level immune pressures, sequences during an early pandemic period (on or before 31 December 2020; n = 525,958) and a later pandemic period (1 July 2022 to 31 December 2024; n = 148,957) were downloaded from the GISAID database. Only human-derived, complete, high-coverage sequences were included in the analysis. Spike protein sequences were analyzed using Nextclade v3.18.1 [39].

### Antibody cocktail suppresses viral escape

To evaluate whether combining S2′-helix-targeting antibodies with stem-helix antibodies prevents viral escape from stem-helix antibodies, rVSV-S was serially passaged under antibody selective pressure using a CPE-based selection strategy adapted from previously described antibody-selection and CPE-based passaging approaches [24,34]. Briefly, rVSV-S was pre-incubated with serially diluted antibodies for 1 h in DMEM containing 2% FBS. The virus-antibody mixtures were then transferred to pre-seeded Vero E6-ACE2 cells and incubated for 48 h. CPE was graded on a four-point scale (0–4) by comparing with the control wells, as previously reported [24,40]. The grading criteria were defined as follows: 0, no CPE; 1, 0–25%; 2, 25–50%; 3, 50–75%; and 4, 75–100%. For each passage, viral populations from wells showing >50% CPE at the highest antibody concentration were harvested and used for the subsequent passage. This selection process was repeated for 13 consecutive rounds under continued antibody pressure. In the final passage cycle, viral populations showing >50% CPE at the highest antibody concentration were collected and titrated for downstream neutralization assays.

### Structural alignment and in silico mutagenesis

The following crystal structures were retrieved from the PDB for mutational analysis: 76E1-S2′-helix (PDB: 7X9E), C77G12-S2′-helix (PDB: 7U0A), COV44–62-S2′-helix (PDB: 8D36), CC95.108-stem helix (PDB: 8DGW), CC25.106-stem helix (PDB: 8DGU), CC40.8-stem helix (PDB: 7SJS), and CV3–25-stem helix (PDB: 7RAQ). All structural analyses including structural alignment and in silico mutagenesis were performed using Schrödinger Suite (2024–1 release), with visualizations created in PyMOL (Educational Version v2.5). Specifically, because the 8DGW structure contains the HCoV-HKU1 spike stem helix rather than its SARS-CoV-2 counterpart, the HCoV-HKU1 stem helix in 8DGW was first structurally aligned onto the SARS-CoV-2 stem helix in 8DGU using tools implemented in Schrödinger Suite before in silico mutagenesis analysis. In contrast, for the other antibodies, published antibody–SARS-CoV-2 spike complex structures were available in the PDB, and in silico mutagenesis analyses were therefore performed directly based on these structures. Escape mutations were introduced in silico by residue substitution in the corresponding antibody–epitope complex structures. The resulting models were inspected to assess potential local steric effects and changes in antibody–epitope contacts.

### Statistics

All statistical analyses were performed using GraphPad Prism 9.5.0 (GraphPad Software, San Diego, CA). The IC_50_ values for each antibody were determined by nonlinear regression analysis. Data in all figures are presented as mean ± SEM (standard error of the mean).

## Supporting information

S1 Table Identification of CC95.108- and CC40.8-selected escape mutations in SARS-CoV-2 by next-generation sequencing (NGS). (DOCX)

S1 Fig Sequence alignment of the S2′-helix and stem helix regions across variants under monitoring (VUMs), variants of interest (VOIs), and variants of concern (VOCs). (DOCX)

S2 Fig Inhibition of spike-mediated membrane fusion by stem helix–targeting antibodies against escape mutants D1153G, Y1155S, F1156L and F1156V. (DOCX)

S3 Fig Inhibition of spike-mediated membrane fusion by stem helix–targeting antibodies against mutants in residue D1153. (DOCX)

S4 Fig Inhibition of spike-mediated membrane fusion by S2′-helix–targeting antibodies against mutant L822F. (DOCX)

S5 Fig Expression, membrane fusion, and viral entry properties of SARS-CoV-2 spike mutants. (DOCX)

S1 File Uncropped western blot images corresponding to S5 Fig. (DOCX)
