# Supplementary material for: The SARS-CoV-2 S2′-helix compared to stem helix confers higher genetic barrier to antibody resistance
Source: PLoS Pathog. 2026 Aug 4;22(8):e1014497. doi: 10.1371/journal.ppat.1014497 (PMC13466045; doi:10.1371/journal.ppat.1014497)
Supplement: Supplementary file 1 [file ppat.1014497.s001.docx]

**S1 Table. Identification of CC95.108- and CC40.8-selected escape mutations in SARS-CoV-2 via next-generation sequencing.**

Escape mutations selected under pressure from CC95.108 or CC40.8 were identified by next-generation sequencing. For each antibody, two replicates (A and B) were established. Passage-matched controls without antibody selection were also sequenced. Mutations with a frequency ≥5% are displayed.
