# Supplementary material for: The SARS-CoV-2 S2′-helix compared to stem helix confers higher genetic barrier to antibody resistance
Source: PLoS Pathog. 2026 Aug 4;22(8):e1014497. doi: 10.1371/journal.ppat.1014497 (PMC13466045; doi:10.1371/journal.ppat.1014497)
Supplement: Supplementary file 2 [file ppat.1014497.s002.docx]

**S1 Fig. Sequence alignment of the S2’-helix and stem helix epitopes among SARS-CoV-2 variants under monitoring (VUMs), variants of interest (VOIs), and variants of concern (VOCs).**

Critical epitope residues are marked with an asterisk (*), dots indicate identity residues with Wuhan-Hu-1.
