# Supplementary material for: The SARS-CoV-2 S2′-helix compared to stem helix confers higher genetic barrier to antibody resistance
Source: PLoS Pathog. 2026 Aug 4;22(8):e1014497. doi: 10.1371/journal.ppat.1014497 (PMC13466045; doi:10.1371/journal.ppat.1014497)
Supplement: Supplementary file 3 [file ppat.1014497.s003.docx]

**S2 Fig.** **Inhibition of spike-mediated membrane fusion by stem helix–targeting antibodies against escape mutants D1153G, Y1155S, F1156L and F1156V.**

**(A)** Schematic of the Cre/Stop-Luc cell–cell fusion assay. Effector cells expressing SARS-CoV-2 spike and Cre recombinase are co-cultured with target cells expressing hACE2 and a loxP-STOP-loxP luciferase reporter. Spike–ACE2-mediated fusion triggers Cre excision of the STOP cassette, activating luciferase expression quantified by luminescence. Created in BioRender. Xie, W. (2026) https://BioRender.com/b59q2pf **(B)** Fusion inhibition by stem helix–targeting antibodies. Escape mutants D1153G and F1156L were selected under CC95.108 pressure; Y1155S and F1156V were selected under CC40.8 pressure. Effector cells expressing spike and Cre were co-cultured with target cells in the presence of CC95.108, CC40.8, or CV3-25 at 20 and 4 μg/mL. Fusion inhibition is shown as percent inhibition relative to the no-antibody control for each spike variant. Data represent mean ± SEM from triplicate wells and are representative of two independent experiments. Statistical analysis was performed by multiple t tests to compare each mutant with WT at the same antibody concentration. * *P* < 0.05, ** *P* < 0.01, *** *P* < 0.001, **** *P* < 0.0001.
