# Supplementary material for: The SARS-CoV-2 S2′-helix compared to stem helix confers higher genetic barrier to antibody resistance
Source: PLoS Pathog. 2026 Aug 4;22(8):e1014497. doi: 10.1371/journal.ppat.1014497 (PMC13466045; doi:10.1371/journal.ppat.1014497)
Supplement: Supplementary file 4 [file ppat.1014497.s004.docx]

**S3 Fig.** **Inhibition of spike-mediated membrane fusion by stem helix–targeting antibodies against mutants in residue D1153.**

Effector cells expressing SARS-CoV-2 spike (WT or indicated mutants) and Cre were co-cultured with hACE2/Stop-Luc target cells in the presence of antibodies (CC95.108, CC40.8, or CV3-25) at the indicated concentrations (20 and 4 μg/mL). Fusion inhibition is expressed as percent inhibition relative to the no-antibody condition for each Spike variant. Bars represent mean ± SEM from triplicate wells. Data shown are representative of two independent experiments. Statistical analysis was performed by multiple t tests to compare each mutant with WT at the same antibody concentration. * *P* < 0.05, ** *P* < 0.01, *** *P* < 0.001, **** *P* < 0.0001.
