# Supplementary material for: The SARS-CoV-2 S2′-helix compared to stem helix confers higher genetic barrier to antibody resistance
Source: PLoS Pathog. 2026 Aug 4;22(8):e1014497. doi: 10.1371/journal.ppat.1014497 (PMC13466045; doi:10.1371/journal.ppat.1014497)
Supplement: Supplementary file 5 [file ppat.1014497.s005.docx]

**S4 Fig.** **Inhibition of spike-mediated membrane fusion by S2’-helix–targeting antibodies against mutant L822F.**

Effector cells expressing SARS-CoV-2 spike (WT or L822F) and Cre were co-cultured with hACE2/Stop-Luc target cells in the presence of antibodies (76E1, C77G12, or COV44-62) at the indicated concentrations (100 and 33 μg/mL). Fusion inhibition is expressed as percent inhibition relative to the no-antibody condition for each spike variant. Bars show mean ± SEM from triplicate wells. Data shown are representative of two independent experiments. Statistical analysis was performed by multiple t tests to compare each mutant with WT at the same antibody concentration. ** *P* < 0.01; ns, not significant.
