# Supplementary material for: The SARS-CoV-2 S2′-helix compared to stem helix confers higher genetic barrier to antibody resistance
Source: PLoS Pathog. 2026 Aug 4;22(8):e1014497. doi: 10.1371/journal.ppat.1014497 (PMC13466045; doi:10.1371/journal.ppat.1014497)
Supplement: Supplementary file 6 [file ppat.1014497.s006.docx]

**S5 Fig. Expression, membrane fusion, and viral entry properties of SARS-CoV-2 spike mutants.**

**(A)** Representative Western blot showing spike protein expression in WT and mutant constructs, with β-actin as the loading control. **(B)** Spike-mediated membrane fusion activity measured using a Cre/Stop-Luc cell–cell fusion assay. Effector cells expressing SARS-CoV-2 spike and Cre recombinase are co-cultured with target cells expressing hACE2 and a loxP-STOP-loxP luciferase reporter, and fusion activity was quantified by luminescence. Data are shown as mean ± SEM from triplicate wells. **(C)** Viral entry of SARS-CoV-2 pseudoviruses carrying individual mutations in Caco-2 cells, quantified by luminescence. Data are shown as mean ± SEM from triplicate wells.
