# Supplementary material for: The SARS-CoV-2 S2′-helix compared to stem helix confers higher genetic barrier to antibody resistance
Source: PLoS Pathog. 2026 Aug 4;22(8):e1014497. doi: 10.1371/journal.ppat.1014497 (PMC13466045; doi:10.1371/journal.ppat.1014497)
Supplement: Supplementary file 7 [file ppat.1014497.s007.docx]

**S1 Rawgel. Uncropped western blot images corresponding to S5 Figure.**

Uncropped western blot images showing the detection of the SARS-CoV-2 S protein (left) and β-actin (right).
